# Detection of Brain Network Communities During Natural Speech Comprehension From Functionally Aligned EEG Sources

**DOI:** 10.3389/fncom.2022.919215

**Published:** 2022-07-07

**Authors:** Di Zhou, Gaoyan Zhang, Jianwu Dang, Masashi Unoki, Xin Liu

**Affiliations:** ^1^School of Information Science, Japan Advanced Institute of Science and Technology, Ishikawa, Japan; ^2^College of Intelligence and Computing, Tianjin Key Laboratory of Cognitive Computing and Application, Tianjin University, Tianjin, China

**Keywords:** community detection, neural entrainment, temporal response function (TRF), source localization, electroencephalography

## Abstract

In recent years, electroencephalograph (EEG) studies on speech comprehension have been extended from a controlled paradigm to a natural paradigm. Under the hypothesis that the brain can be approximated as a linear time-invariant system, the neural response to natural speech has been investigated extensively using temporal response functions (TRFs). However, most studies have modeled TRFs in the electrode space, which is a mixture of brain sources and thus cannot fully reveal the functional mechanism underlying speech comprehension. In this paper, we propose methods for investigating the brain networks of natural speech comprehension using TRFs on the basis of EEG source reconstruction. We first propose a functional hyper-alignment method with an additive average method to reduce EEG noise. Then, we reconstruct neural sources within the brain based on the EEG signals to estimate TRFs from speech stimuli to source areas, and then investigate the brain networks in the neural source space on the basis of the community detection method. To evaluate TRF-based brain networks, EEG data were recorded in story listening tasks with normal speech and time-reversed speech. To obtain reliable structures of brain networks, we detected TRF-based communities from multiple scales. As a result, the proposed functional hyper-alignment method could effectively reduce the noise caused by individual settings in an EEG experiment and thus improve the accuracy of source reconstruction. The detected brain networks for normal speech comprehension were clearly distinctive from those for non-semantically driven (time-reversed speech) audio processing. Our result indicates that the proposed source TRFs can reflect the cognitive processing of spoken language and that the multi-scale community detection method is powerful for investigating brain networks.

## 1. Introduction

Speech comprehension is the acquisition of communicative information from speech sounds, which links auditory stimuli and cognitive processes (Zhang et al., 2019). During speech comprehension, low-level uninterrupted acoustic features are transferred to high-level linguistic information, which in turn is integrated into meaningful sentences and also stories (Gaskell and Mirkovic, 2016). One of the main objectives of auditory neuroscience is to investigate how the human brain comprehends perceived speech. In past decades, researchers tried to use isolated words or simple sentences to investigate speech comprehension in the human brain. In those studies, subjects were asked to participate in specific tasks such as identifying whether the perceived word is a real word or a pseudoword (Binder et al., 2009; Zhang et al., 2019), or assessing whether a word in a sentence is congruent or incongruent with the rest of the sentence (Chow and Phillips, 2013). With this kind of well-designed paradigm, researchers can use a statistical analysis method (such as *t*-tests, analysis-of-variance) to estimate the mechanism of speech processing by comparing neural behaviors between different conditions. For example, in an experiment designed to study the N400 amplitude and word expectancy, it was found that N400s to sensible and equally low-cloze-probability completions of strongly constraining sentences (e.g., The bill was due at the end of the hour) were much larger than those to high-cloze-probability endings (The bill was due at the end of the month) (Kutas and Federmeier, 2011). However, such a task is far away from human speech comprehension in daily life. In recent years, studies have extended the controlled experimental paradigm, from isolated words or simple sentences to a more natural experimental setting, such as listening to continuous speech with a complete storyline (Brennan, 2016). Under the natural language paradigm, previous studies treat the neural system as a linear time-invariant (LTI) system. Although it is too rough to treat the complicated neural system as an LTI system, the reversible properties of the LTI system are helpful for evaluating the performance of the linearized neural system. The impulse response of the neural system is referred to as a temporal response function (TRF) hereafter. The TRF can describe the neural response from a speech stimulus, which can be represented by a speech envelope, phoneme sequence, or a spectrogram, to a specific electrode of the EEG setting, which is also called neural entrainment to speech (Ding and Simon, 2012; Brodbeck et al., 2018b; Pan et al., 2019). Many studies have proved that TRFs can reflect high-level language processing to some extent, such as categorical representations of phonemes (Liberto et al., 2015), perception attention and speech comprehension processing (Broderick et al., 2018; Weissbart et al., 2020), and lexical processing (Brodbeck et al., 2018a). Because most language cognitive processes occur within hundreds of milliseconds, high temporal resolution electroencephalograph/magnetoencephalography (EEG/MEG) techniques are often used for the natural spoken language paradigm.

Although EEG/MEG is an effective, non-invasive technique for investigating the neural mechanism behind auditory processing, due to the low spatial resolution of EEG/MEG, most previous studies investigate natural spoken language only in the electrode/sensor space (Liberto et al., 2015; Broderick et al., 2018; Etard and Reichenbach, 2019). Because the signal of an EEG electrode is a mixture of many source components, previous research cannot explain the cortical origins of the underlying natural spoken language processes. With the development of EEG signal processing, it has been proved that with enough sensors or using an accurate individual head model with reasonable conductivity values, EEG source localization may be precise enough to reflect the cortical origins of language processing (Klamer et al., 2015). In addition, as generators of neural activity cannot unambiguously be interpreted from sensor EEG data, which cannot provide useful information to explore the underlying brain mechanism, study done in the source space is beneficial to explicitly exploring brain functions in response to continuous speech (Stropahl et al., 2018). Therefore, this study investigates the neural responses to continuous speech on the basis of sources reconstructed from EEG signals.

More recent studies got some exciting results for both speech production and speech perception based on EEG source localization techniques (Stropahl et al., 2018; Zhang et al., 2019; Janssen et al., 2020). However, most of them are based on event-related potential (ERP) paradigm (Handy, 2005). In particular, in the natural speech paradigm, stimuli are typically long segments from lectures or stories and presented to subjects only once to avoid a priming effect. There are two key problems that need to be solved for single-trial paradigm before source localization. First, It is acknowledged that the generated electrical fields are easily contaminated by external noise (e.g., eye movement, head movement) that occurs during the transmission from the neural population to the top layer of the scalp through the brain tissue and skull. If we reconstruct a source single from a single trial and then fit TRFs to cortical sources directly, the unexpected noise may affect the accuracy and interpretability of source-based TRFs. In addition, most of the recent source localization techniques are developed for the ERP paradigm based on the assumption of spatiotemporal sparsity (Grech et al., 2008; Pirondini et al., 2017; Mannepalli and Routray, 2018; Liu et al., 2019; Asadzadeh et al., 2020). However,he natural speech paradigm does not allow for additive averaging across repeated trials common in the source localization of responses evoked from EEG. To solve the problem, we propose using additive averaging across subjects to improve the accuracy of source localization. Assuming that the brain functions for speech processing are consistent across individuals, a similar neural response can be expected from different subjects for the same speech stimulus. In contrast, external noise, involuntary breathing, and attentiveness differ from individual to individual, and such noises can be suppressed by averaging the neural signals of the same stimuli for all subjects. However, this is difficult due to the lack of methods that account for subjects' differences in terms of the setup positions of the electrodes. Addressing this problem well before averaging neural activities across multiple subjects should result in more accurate source localization.

To do so, we first propose a functional hyper-alignment method to reduce the mismatching caused by individual experiment settings, and source reconstruction is then performed on the basis of additive averaging over all subjects for each trial. TRFs are then estimated independently for each localized brain source; they are then related to one another by using Pearson correlation to construct the whole brain functional network. Previous research proved that the brain network can be characterized by its community structure, and community detection for functional brain networks has facilitated the understanding of the underlying brain organization and its related cognitive function (Forbes et al., 2018). Therefore, we use the community detection method to detect the community structure of the brain networks underlying continuous speech comprehension. We compared the community organization between the understanding of a naturally told story and a time-reversed story to study the brain mechanism related to semantic processing underlying continuous speech comprehension.

## 2. Methods

### 2.1. Noise Reduction by Additive Averaging

There are many kinds of external noises caused by eye movement, heartbeat, electrical noise, and so on. They occur during the transmission from the neural population to the scalp through the brain tissue and skull and are mixed into EEG signals (Cohen, 2014). Supposing these noises are random, ERP analysis removes these kinds of noises by applying an addition operation to a number of trials for the same task, namely additive averaging. Our study adopts an idea similar to that used in the ERP technique to reduce noise, but we apply additive averaging to EEG signals for the same stimulus material over all subjects since subjects can listen to the same material only once. In our previous study, we proved that additive averaging across subjects can improve the accuracy of TRFs (Di Zhou et al., 2020).

To apply additive averaging across subjects, it is better to calibrate the individual differences in the setup positions of the electrodes. To tackle this problem, we propose a functional hyper-alignment method for soft calibration. It uses a well-designed spatial filter to align the setup positions of electrodes by minimizing the distance of the signal features among the subjects. Due to the lack of methods that account for subjects' differences in the setup stage of the electrodes, the position of an electrode *n* for subject *i* may not be the same as that of subject *j*. Thus, the additive average over the EEG data *x*_*i*_(*t, n*)(*i* = 1, 2, ..., *I*) cannot be used to perform denoising properly, where *I* is the subject number. For this reason, we propose using a functional hyper-alignment method for eliminating this effect. The main idea of the functional hyper-alignment is to rotate *x*_*i*_(*t, n*) and *x*_*j*_(*t, n*) (*i* ≠ *j* ∈ [1, 2, ..., *I*]) to maximize their correlation among subjects. So far, several methods have been proposed for this purpose, such as group task-related component analysis (gTRCA) (Tanaka, 2020) and multi-set canonical correlation analysis (MCCA) (de Cheveigné et al., 2019). We choose MCCA to maximize the data correlation among subjects, which satisfies the requirement of our study.

The goal of MCCA is to find projection vectors ω that maximize the correlation between multiple data sets *X*_*i*_,*i* = 1, 2..., *I*. The correlation ρ of all data sets can be calculated as the ratio of the summations of the between-set covariance *V*_*x*_*i*_*x*_*j*__ over the within-set covariance *V*_*x*_*i*_*x*_*i*__,


(1) $$\begin{array}{l} p\left({\tilde{X}}_{1},{\tilde{X}}_{2},...,{\tilde{X}}_{i},...,{\tilde{X}}_{I}\right)=\frac{1}{N-1}\frac{\sum_{i=1}^{I}\sum_{j=1,i\neq j}^{I}\omega_{i}^{T}V_{x_{i}x_{j}}\omega_{j}}{\sum_{i=1}^{I}\omega_{i}^{T}V_{x_{i}x_{i}}\omega_{i}}, \end{array}$$

where


(2) $$\begin{array}{l} V_{x_{i}x_{j}}={\left(X_{i}-{\overset{-}{X}}_{i}\right)}^{T}\left(X_{j}-{\overset{-}{X}}_{j}\right), \end{array}$$

(3) $$\begin{array}{l} V_{x_{i}x_{i}}={\left(X_{i}-{\overset{-}{X}}_{i}\right)}^{T}\left(X_{i}-{\overset{-}{X}}_{i}\right). \end{array}$$

${\overset{-}{X}}_{i}$, ${\overset{-}{X}}_{j}$ are the means for set *i* and set *j*. $\frac{1}{N-1}$ ensures that the correlation ρ scales between 0 and 1. Altogether, the above equation can be summarized into a generalized eigenvalue problem,


(4) $$\begin{array}{l} B\omega =\lambda R\omega , \end{array}$$

where


(5) $$\begin{array}{l} B=\left[\begin{array}{cccc} O & V_{x_{1}x_{2}} & \cdots & V_{x_{1}x_{I}}\\ V_{x_{2}x_{1}} & O & \cdots & V_{x_{2}x_{I}}\\ \vdots & \vdots & \ddots & \vdots \\ V_{x_{I}x_{1}} & V_{x_{I}x_{2}} & \cdots & O \end{array}\right],R=\left[\begin{array}{cccc} V_{x_{1}x_{1}} & O & \cdots & O\\ O & V_{x_{2}x_{2}} & \cdots & O\\ \vdots & \vdots & \ddots & \vdots \\ O & O & \cdots & V_{x_{I}x_{I}} \end{array}\right]. \end{array}$$

*B* is a matrix combining all between-set covariance *V*_*x*_*i*_*x*_*j*__, and *R* is a diagonal matrix that contains all within-set covariance *V*_*x*_*i*_*x*_*i*__. ω is a spatial vector set for an entire data set $\omega =\left[\omega_{1}^{T},\omega_{2}^{T},\ldots ,\omega_{I}^{T}\right]$. Finally, the spatial filter for aligning the positions of the electrodes is reduced to solve the generalized eigenvalue problem.

### 2.2. Source Reconstruction Based on Denoising EEG Data

After the denoising, the EEG data are used to estimate their cortical source activations in the brain, namely source reconstruction. In this study, the forward and reverse models for source localization were calculated by the Brainstorm toolbox (Tadel et al., 2011). The finite element method (FEM) as implemented in DUNEuro was used to compute the forward head model using Brainstorms default parameters with an MNI MRI template (ICBM152) (Vorwerk et al., 2016; Schrader et al., 2021). The FEM models provide more accurate results than the spherical forward models and more realistic geometry and tissue properties than the boundary element method (BEM) methods (Gramfort et al., 2010). For source estimation, the number of potential sources (grid on the cortex surface) is set to 15,002. And the option of constrained dipole orientations was selected, which means dipoles are oriented perpendicular to the cortical surface (Tadel et al., 2011). We then apply the method of standardized low-resolution electromagnetic tomography (sLORETA) (Pascual-Marqui, 2002) to obtain plausible EEG source estimates. Although the spatial resolution of sLORETA is low, sLORETA can provide smooth and good localization with few localization errors (Asadzadeh et al., 2020). Finally, according to the Desikan-Killiany Atlas (DKA), the cortical surface is divided into 68 anatomical regions of interest (ROIs) (Desikan et al., 2006). The time series of each ROI is calculated from the average value of all dipoles in the respective region. As a result, we obtain a series of brain areas (sources) that are activated in speech comprehension.

### 2.3. Source-Based TRFs Estimation

To evaluate the brain functions explicitly, we calculate the TRFs from speech input to sources in the brain cortex, instead of the electrodes on the scalp. The mTRF toolbox (https://github.com/mickcrosse/mTRF-Toolbox) is used to linearly map the speech envelope and the neural response in the sources by approximating the brain as an LTI system (Crosse et al., 2016). Let $\hat{r}\left(\tau ,ROI_{n}\right)$ be the TRF of a brain region *ROI*_*n*_ for an input speech envelope *s*(*t*), the neural response signal $\hat{x}\left(t,ROI_{n}\right)$ of the source *ROI*_*n*_ can be described as follows.


(6) $$\begin{array}{l} \hat{x}\left(t,ROI_{n}\right)=\underset{\tau }{\sum }\hat{r}\left(\tau ,ROI_{n}\right)s\left(t-\tau \right) \end{array}$$

The range for τ is from 0 to 800 ms in our study, as most common ERP components in language research are within 800 ms (Beres, 2017). The broadband temporal speech envelopes of *s*(*t*) are obtained from a gammatone filterbank followed by a power law (Biesmans et al., 2016; Peng et al., 2018, 2021). For the modeling process, the envelope is decimated to the same sampling rate as the source signal, enabling us to relate its dynamics to the source.

Under the theory for the LTI system, the backward approach can be modeled using a decoder ${\hat{r}}^{-1}\left(\tau ,ROI_{n}\right)$, which is the inverse function of $\hat{r}\left(\tau ,ROI_{n}\right)$. The optimal decoder ${\hat{r}}^{-1}\left(\tau ,ROI_{n}\right)$ is acquired by minimizing the MSE between the original and predicted speech envelope, and *n* denotes the number of regions. Thus, the input speech stimulus *s*(*t*) can be decoded from the source neural signal $\hat{x}\left(t,ROI_{n}\right)$ using the decoder function ${\hat{r}}^{-1}\left(\tau ,ROI_{n}\right)$. This can be expressed as follows.


(7) $$\begin{array}{l} s\left(t\right)=\underset{n}{\sum }\underset{\tau }{\sum }{\hat{r}}^{-1}\left(\tau ,ROI_{n}\right)\hat{x}\left(t-\tau ,ROI_{n}\right) \end{array}$$

The encoder $\hat{r}\left(\tau ,ROI_{n}\right)$ and decoder ${\hat{r}}^{-1}\left(\tau ,ROI_{n}\right)$ approach the optimal values when iterating the above calculation.

### 2.4. Brain Network Analysis Based on Community Detection Method

#### 2.4.1. Construction of Preliminary Brain Network

The brain network can be characterized as a community structure. Therefore, community detection is often used in exploring the brain network during a given task (Jin et al., 2019). To do so, we first need to define the nodes of the brain and links of the network (Yu et al., 2018). In large-scale brain networks, nodes usually represent brain regions, and links represent anatomical, functional, or effective connections (Friston et al., 1994). The pre-defined spatial regions of interest (ROIs) assessed by anatomical atlases are one of the most popular methods for defining brain nodes (Smith, 2012). This study uses the 68 nodes (brain region) that were defined in the DKA, and it uses Pearson correlation to describe the functional link among the nodes (Smith et al., 2013). This would result in 2,278 $\left(=C_{68}^{2}\right)$ edges if linking all pairwise nodes for each trial. Differing from the previous studies, the link weights (temporal correlations) here are calculated using the TRF of each node, but not the source neural signal. As a result, we obtain a preliminary brain network that consists of all of the brain regions and pairwise links with a weighted edge.

#### 2.4.2. Community Detection in Functional Brain Networks

Community detection divides the nodes of the preliminary network into a number of non-overlapping clusters and then detects the communities in a functional network by maximizing the module quality metric *Q* (Newman and Girvan, 2004), which is also called modularity. A higher value of modularity represents that the detection approaches a more evident community structure. Therefore, this algorithm provides not only a community partition but also an index for evaluating whether a network community structure is evident. Recent studies have proposed that the negative correlation in the functional connection matrix also possesses some physiological significance, and correlated and anti-correlated brain activities may signify cooperative and competitive interactions between brain areas that subserve adaptive behaviors (Khambhati et al., 2018; Zhang and Liu, 2021). Therefore, we used an optimized community detection algorithm in the Brain Connectivity Toolbox (Anwar et al., 2016) on a preliminary connection matrix to detect the community structure of the functional connection matrix for different densities, which takes into account both positive and negative correlations in a network (Bolton et al., 2018; Zhang and Liu, 2021). Since the density as a threshold reflects how many edges are effective in a network, density selection also has a significant impact on studying brain function (Liu et al., 2011; Jrad et al., 2016; Jin et al., 2019). To determine the brain network more accurately, this study uses different scales to divide the brain network; thus, it can detect the optimal communities of a brain network using different densities. While running this algorithm, the default resolution parameter of γ is set to 1, yielding modularity scores *Q*_*d*_ and density *D*_*t*_ for *t* = 0.01, 0.02, ...., 0.1, ...., 0.5, where *t* is the variation of density.

#### 2.4.3. Brain Network Selection

For community detection with different scales, it is important to find the optimal functional brain networks from the scales. The scale means the different thresholds which can sparse the brain networks in different densities. On the basis of a previous study (Jin et al., 2019), this study uses the variation of information (VI) to evaluate the similarity of community structures with different scales in functional brain networks. VI can compare two community structures by means of their information exchange loss and gain. VI can be described as


(8) $$\begin{array}{l} VI\left(X,Y\right)=H\left(X\right)+H\left(Y\right)-2I\left(X;Y\right), \end{array}$$

where *X* and *Y* are two different community structures of the brain network. *H*(*X*) and *H*(*Y*) are the entropy for *X* and *Y*, respectively. *I*(*X*; *Y*) is the mutual information between *X* and *Y*.

When VI is equal to zero, it represents the most stable partition across densities (He et al., 2018). According to VI values, we can obtain the most reasonable community partition of functional brain networks. In addition to VI, this study also uses cluster analysis on the different scales to find the best discrimination for separating natural speech and time-reversed speech in brain networks. Finally, we evaluate the selected brain network using the current novel results of brain research (Walenski et al., 2019).

## 3. Experiments

### 3.1. Participants

Twenty-four healthy Mandarin Chinese speakers (mean ± standard deviation age, 22 ± 2.4 years; nine males; right-handed) were recruited from Tianjin University and Tianjin University of Finance and Economics. The experiments were conducted in accordance with the Declaration of Helsinki (World Medical Association, 2014) and were approved by the local ethics committee. The subjects signed informed consent forms before the experiment and were paid for their participation afterward. All the subjects reported no history of hearing impairment or neurological disorders.

### 3.2. Materials and Experimental Procedure

Subjects undertook 48 non-repetitive trials. Among them, 24 trials were short stories (around 60 s) with a complete storyline, recorded by a male Chinese announcer in a soundproof room. The other 24 trials were the same story segments but were time reversed. All stimuli were mono speech with a 44.1 kHz sampling rate, and the stimulus amplitudes were normalized to have the same root mean square (RMS) intensity. The 48 trials were randomly presented to the subjects. All speech segments were also modified to truncate silence gaps to <0.5 s (Brodbeck et al., 2018b).

The experiments were carried out in an electronically and magnetically shielded soundproof room. Speech sounds were presented to subjects through Etymotic Research ER-2 insert earphones (Etymotic Research, Elk Grove Village, IL, USA) at a suitable volume (around 65 dB). During each trial, subjects were instructed to focus on a crosshair mark in the center of the screen to minimize head movements and other bodily movements. There was a 5 s interval between each trial, and the subjects were given a 5 min break every 10 trials. After each story trial, subjects were asked immediately to answer multiple-choice questions about the content of the story to ensure that they focused on the auditory task. We embedded unique tones in some trials to draw more of the subjects' attention to the reversed stimuli. Subjects were requested to detect the tones and indicate how many times they appeared after the trial. The EEG data corresponding to the embedded tones was removed in further analysis.

### 3.3. EEG Data Acquisition and Pre-processing

Scalp EEG signals were recorded with a 128-channel Neuroscan SynAmps system (Neuroscan, USA) at a sampling rate of 1,000 Hz. Six of the channels were used for recording a vertical electrooculogram (VEOG), a horizontal electrooculogram (HEOG), and two mastoid signals. The impedance of each electrode was kept below 5 kΩ during data acquisition. Three subjects' data were discarded in further analysis because they did not give a proper answer to the multiple-choice questions or the electrodes detached during the EEG data recording. The raw EEG data were pre-processed using the EEGLAB toolbox (https://sccn.ucsd.edu/eeglab/index.php) in MATLAB (MathWorks) (Delorme and Makeig, 2004). This involved removing sinusoidal (i.e., line) noise and bad channels (i.e., low-frequency drifts, noisy channels, short-time bursts) and repairing the data segments (Perrin et al., 1989; Plechawska-Wojcik et al., 2018). Then, the EEG data was downsampled to 250 Hz, 1 Hz high-pass filtering was performed to remove linear drift, and 40 Hz low-pass filtering was performed to remove power frequency interference and high-frequency noise. Adaptive mixture independent component analysis (AMICA) (Palmer et al., 2012) and ICLabel (Pion-Tonachini et al., 2019) were used to automatically identify and remove artifact components.

### 3.4. Overview of Proposed Approach

**Figure 1:**
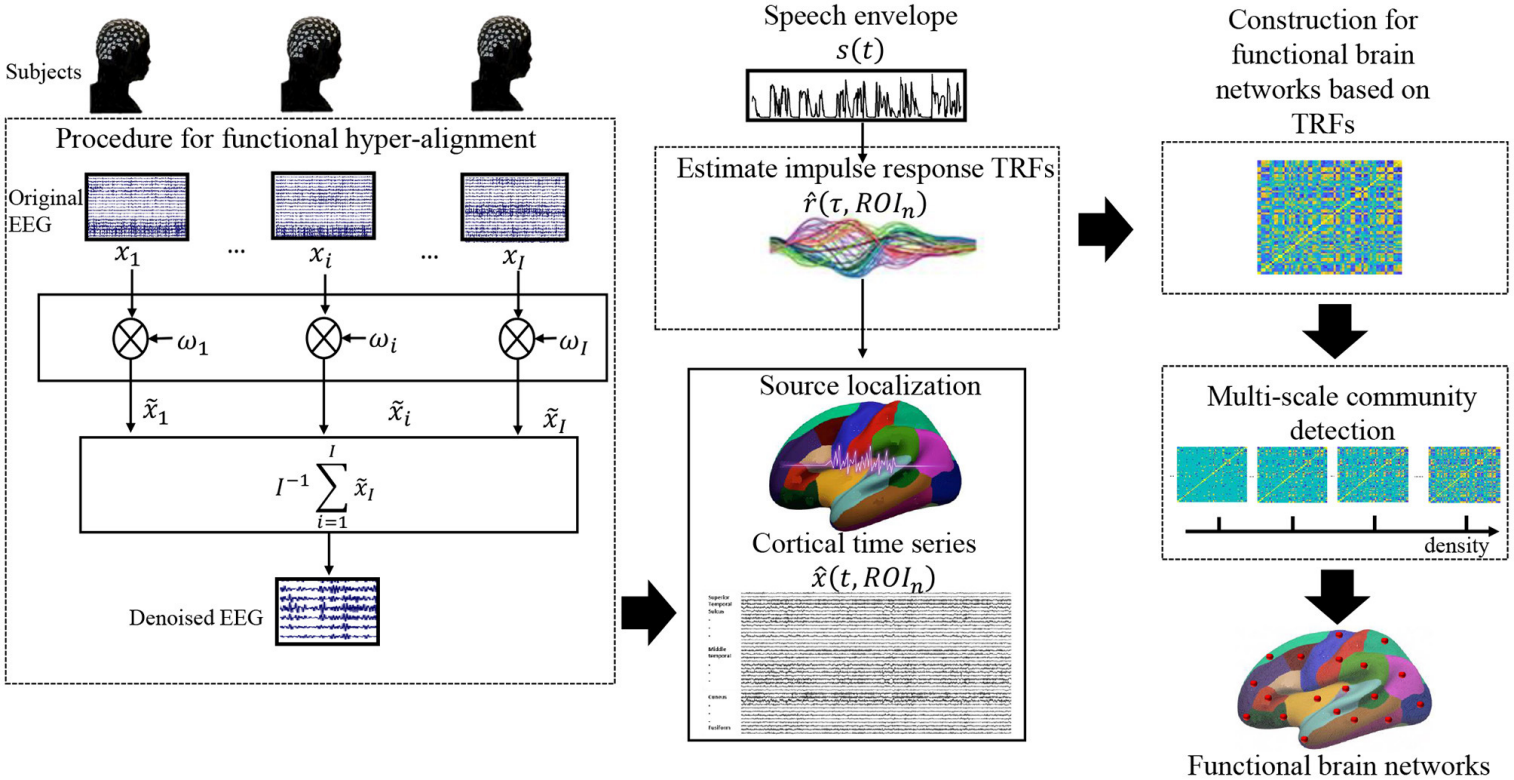
Flowchart of the data processing approaches in this study.

As mentioned above, we reasonably assume that brain functions for processing the same speech material are consistent across individuals; thus, consistent neural responses were expected for all subjects to the same speech stimulus. In contrast, potential noise, eye movement, involuntary breathing, and attentiveness differ from individual to individual as well as the individual electrode setting. To calibrate the electrode positions across subjects, we used functional hyper-alignment and applied it to the EEG data first. After the calibration, we suppressed the random noises by additive averaging of the neural signals of the same stimulus over all subjects. Then, the denoised EEG singles were used to reconstruct the neural sources of EEG data in the brain, and the TRFs for the neural sources were estimated. Using the TRFs, we constructed a functional brain network of natural speech processes on different scales. Finally, an optimal functional brain network was decided on the basis of the VI of the communities with the multiple scales. A flowchart for the proposed approach is shown in Figure 1.

## 4. Results

### 4.1. Accuracy for Source-Based TRFs

#### 4.1.1. Evaluation With Results of Speech Envelope Prediction

**Figure 2:**
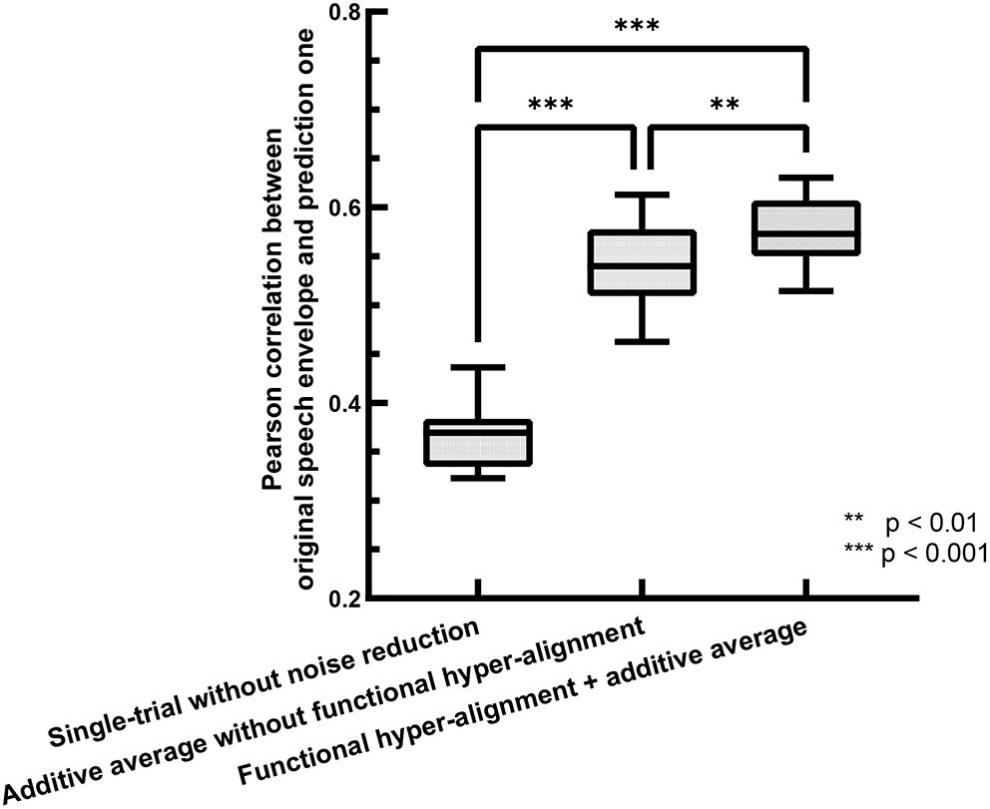
Comparison of envelope prediction accuracies between functional hyper-alignment method and other two methods.

The accuracy of the backward prediction of stimulus input from neural output is usually used for evaluating the performance of TRFs (O'Sullivan et al., 2015; Das et al., 2020). Here, we use the backward prediction approach to evaluate the performance of the proposed functional hyper-alignment approach through comparison with the traditional method with single-trial estimation. For training processes, we used a leave-one-out cross-validation procedure, where 23 trials were used for training, and the remaining one was used for testing in each fold. The prediction accuracy was described by the Pearson correlation coefficient between the predicted speech envelopes and the original ones. Figure 2 shows comparisons of the prediction accuracies for the single-trial method without noise reduction (O'Sullivan et al., 2015), the additive average method over the subjects without functional hyper-alignment (Di Zhou et al., 2020), and the functional hyper-alignment method combined with the additive average method. For a fair comparison, the correlation coefficient was first transformed into a *z*-value by Fisher's z transformation to satisfy a normal distribution (Corey et al., 1998). Then, an analysis-of-variance (ANOVA) of the *z*-values revealed a significant effect on the prediction methods (*F* = 193.53, *p* < 0.001). The results of the ANOVA demonstrated that the prediction accuracy of our proposed method, the functional hyper-alignment method combined with the additive average method, was the highest among the three methods. A post-hoc test for the ANOVA showed that the prediction accuracy was significantly improved by the functional hyper-alignment method, compared with the average without the functional hyper-alignment method (*F* = 9.68, *p* < 0.005) and single-trial method (*F* = 400.78, *p* < 0.001). Therefore, the functional hyper-alignment with the additive average method was used in the following TRF estimation.

#### 4.1.2. Evaluation by Comprehension Behaviors

To evaluate the behaviors of the different methods from a brain function point of view, we used a distinct degree of the brain networks in speech comprehension. We judged whether the methods could or could not distinguish the brain network for the normal speech listening task from that for the time-reversed speech listening task since the latter one tackles non-semantic audio sequences. The method with a higher distinct degree between normal play and reverse play would be the better method for our study. Therefore, we estimated source-based TRFs using the proposed method and the single-trial method, and we then constructed functional brain networks based on the TRFs. The link between two brain regions was expressed by a Pearson correlation ranging from −1 to 1. The higher the correlation, the higher the similarity between the two brain regions, and vice versa.

**Figure 3:**
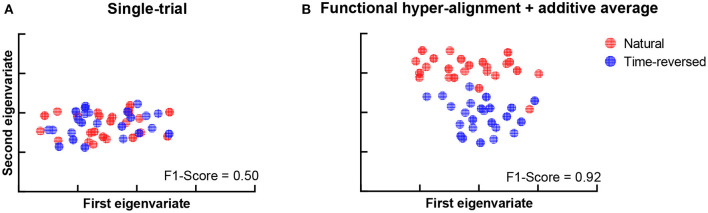
K-means clustering of t-SNE embedded distributions obtained by single-trial method **(A)** and proposed method **(B)**.

During the experiment, we asked the subjects to answer multiple-choice questions about the story presented in the listening task after each trial. The accuracy of the answers of the question was 88.25 ± 4.62% for the normal speech, which indicates that the subjects mostly comprehended it seriously. The speech intelligibility was also evaluated on a numerical rating scale from 1 to 5 by the subjects, where “very easy to understand” was scored 5, and “completely incomprehensible” was scored 1. The speech intelligibility was 4.74 ± 0.45 for the normally played natural story but was 1.46 ± 0.81 for the time-reversed one. This means that speech was not comprehended in the time-reversed case since there was little semantic information. For these reasons, functional brain networks are expected to be separated into two clusters. One cluster is for semantically driven brain activation, and the other is for non-semantically driven audio processing. To clarify our expectation, we used t-distributed stochastic neighbor embedding (t-SNE) (der Maaten and Hinton, 2008) to visualize the brain networks in a two-dimensional representation, and we verified whether or not semantically driven brain activation was separable from non-semantically driven activation. We first reshaped the connection matrix with a size of 68 × 68 to a vector with 2,278 dimensions to analyze the pairwise connections of all the brain nodes for each trial. For 48 trials, we had a matrix with a size of 48 × 2,278, and we applied t-SNE analysis to it. Finally, the results of the t-SNE analysis were clustered by using the k-means algorithm (Liberto et al., 2015). We set the cluster number to 2, and we performed 1,000 k-means repetitions with random initial states. A two-dimensional scatter of the semantically and non-semantically driven brain networks is shown in Figure 3, where the left panel is the result obtained with the single-trial method, and the right panel is for the proposed method. One can see that the functional connections based on our proposed method show distinct clusters for natural and time-reversed speech, while the single-trial method does not show any obvious clusters. The F1-scores of the actual groupings and the k-means clusters were calculated for all repetitions. The average F1-scores of 1,000 repetitions was 0.5 for the single-trial method (Figure 3A) and 0.93 (Figure 3B) for the proposed method. These results indicate that a brain network based on our proposed method can reveal high-level speech comprehension processing; however, that of the single-trial TRF-based brain network cannot.

**Figure 4:**
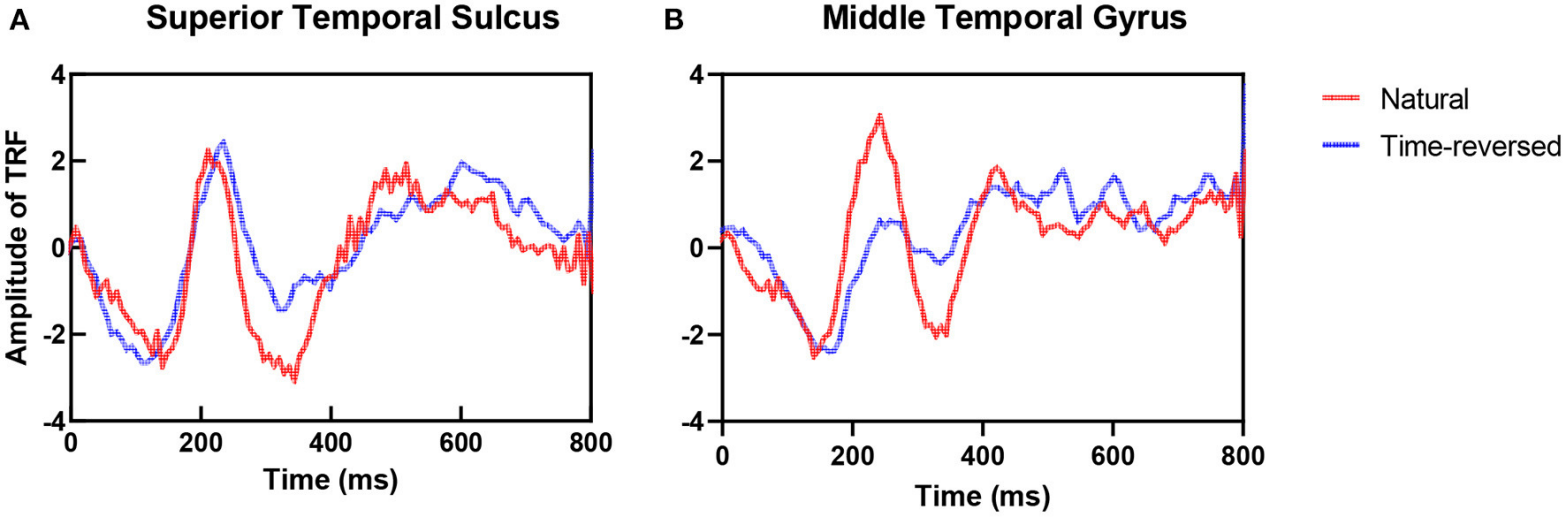
TRFs for natural and time-reversed speech for STS **(A)** and MTG **(B)**.

To address the differences in the brain networks between the natural speech and time-reversed speech in detail, we investigated the difference in the estimated TRFs between the two cases. Figure 4 shows examples of the TRFs of the left superior temporal sulcus (STS) and the left middle temporal gyrus (MTG) for natural speech and time-reversed speech, where STS and MTG are considered to correspond to speech comprehension (Price, 2012). One can see that the patterns of the peaks and troughs for STS (Figure 4A) show a significant difference at time lags between 300 and 450 ms of the TRF (paired *t*-test, *p* = 5.2 × 10^−5^; effect size d = 1). In Figure 4B, the TRF patterns for MTG show a significant difference between 150 and 450 ms (paired *t*-test, *p* = 2.1 × 10^−14^; effect size d = 2).

**Figure 5:**
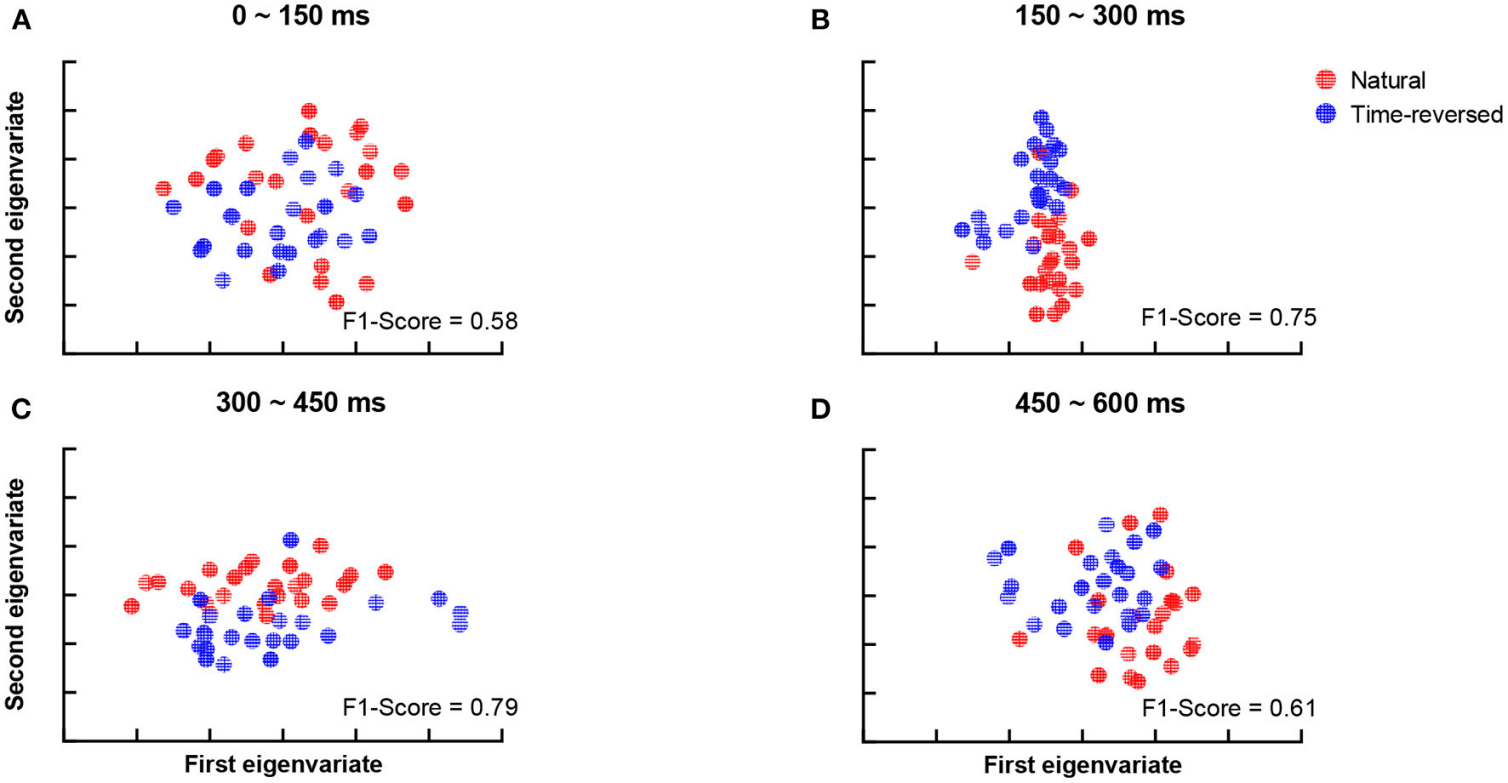
Clustering results for TRF amplitude in different time intervals: 0 ~ 150 ms **(A)**, 150 ~ 300 ms **(B)**, 300 ~ 450 ms **(C)**, 450 ~ 600 ms **(D)**.

According to Beres (2017), the difference in intervals between 150 and 450 ms plausibly corresponds to semantic processing (N400) or syntactic processing (left-anterior negativity, LAN). To verify whether the differences are related to speech comprehension or not, we applied t-SNE and k-means again. To do this, we segment the TRFs into four periods: 0 ~ 150, 150 ~ 300, 300 ~ 450, and 450 ~ 600 ms, and we applied k-means to the amplitudes of the TRFs of the 68 brain regions for each period, respectively. Figure 5 shows the clusters of each period: 0 ~ 150 ms (Figure 5A), 150 ~ 300 ms (Figure 5B), 300 ~ 450 ms (Figure 5C) and 450 ~ 600 ms (Figure 5D). According to the F1-score, one can see that the time between 150 ~ 450 ms (Figure 5C) had the best clustering, which is consistent with the common knowledge that N400 is concerned with semantic processing.

### 4.2. Multi-Scale Community Detection

The investigation above was carried out with a brain network with full connection. As we knew, different speech functions have different brain network communities as speech planning, semantic processing networks. Therefore, some edges in the connection matrix may be invalid or noise for a specific speech process. It is thus necessary to determine the optimal connection matrix on the basis of the different thresholds of the connection.

**Figure 6:**
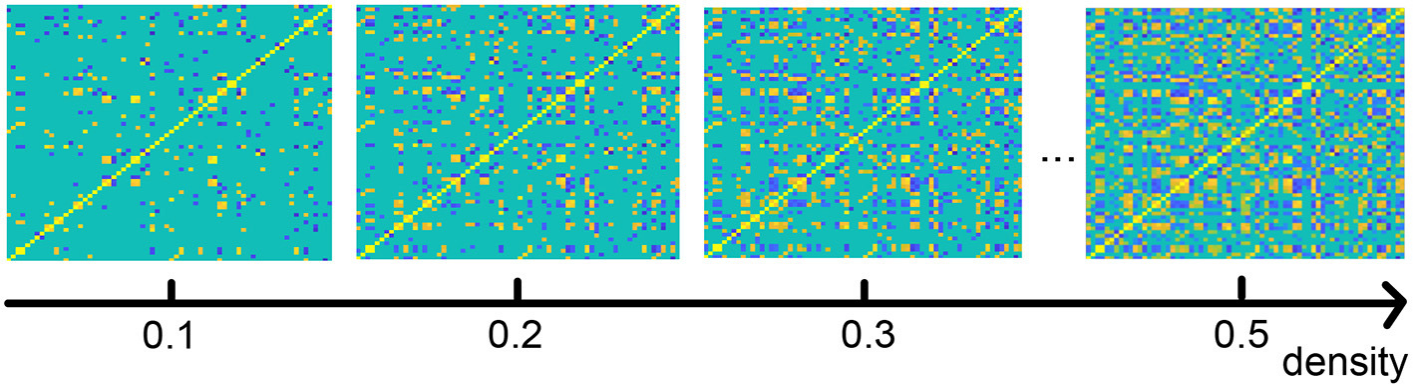
Functional brain connection matrix with different densities. Yellow denotes edge value 1, blue −1.

As described in the section on our method, we analyze the functional connection matrix using different densities, and we investigate the functional brain network at multiple scales (Jin et al., 2019). Figure 6 shows an illustration of the brain connection matrix for four different densities.

**Figure 7:**
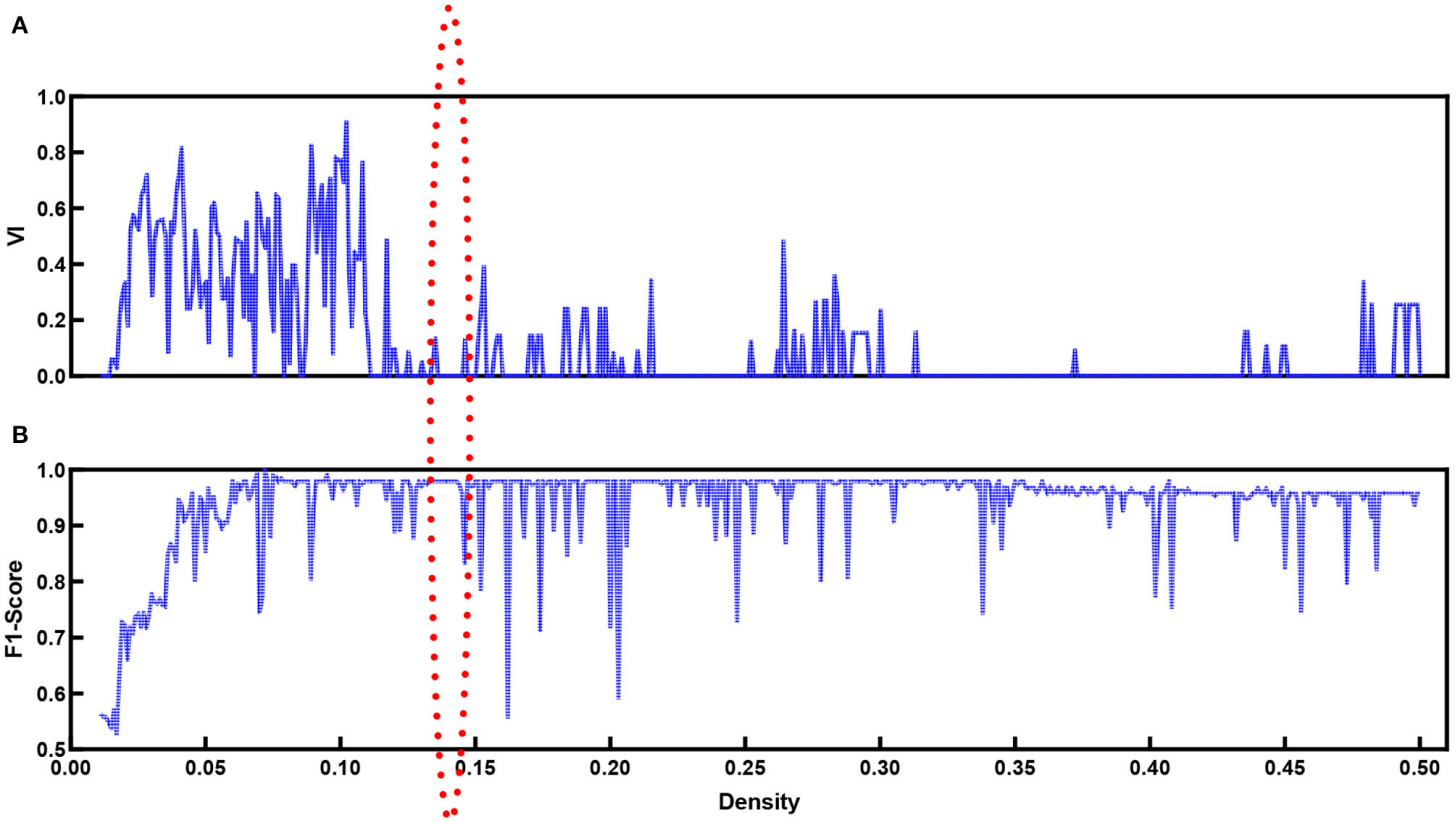
VI **(A)** values and F1-score **(B)** for functional brain network with different density values.

When the modularity *Q* ranges from 0.3 to 0.8, in general, the network contains community structures (Jin et al., 2019). The modularity score *Q*_*d*_ is around 0.64 at any density *D*_*t*_ for the functional brain network based on our proposed method. This implies that community detection is available for our data. In addition to the modularity values, we introduce VI as monitoring variables along with densities to explore the best community division. Figure 7 shows the VI (Figure 7A) along with the density. When the community structure approached a stable situation, the VI value reached the minimum. At the same time, we refer to the F1-score (Figure 7B).

**Figure 8:**
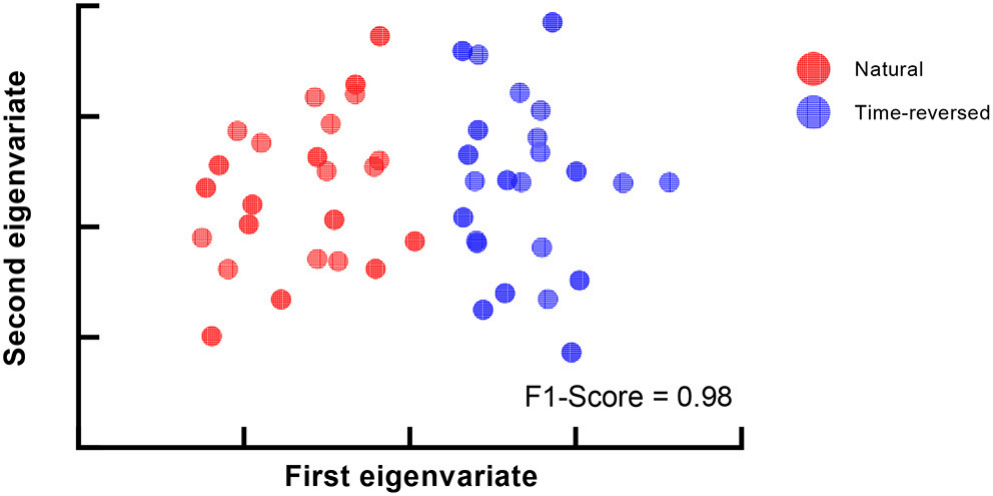
Best clustering results for multi-scale density.

Figure 7B shows the F1-score for k-means used to separate natural and time-reversed speech at different densities of brain networks. Altogether, it seems that the three conditions could be satisfied when the density ranged from 0.136 to 0.143. In this range, we could also obtain the best separation of the brain networks for the natural speech and time-reversed speech, as shown in Figure 8.

In terms of the above investigation, we finally determined the optimal density value to be 0.14, and we obtained community structures using the community detection method. As a result, 16 communities were discovered in the functional brain network during natural speech comprehension, and 19 communities were discovered for the audio processing of the time-reversed speech. From the community detection results, we chose the two largest communities for natural speech and time-reversed speech, respectively, and show them in Table 1. Since the common processing in both cases was audio signal processing, the majority of the brain areas were the same for the natural and time-reversed speech. In the first community, 14 brain regions appeared in both cases simultaneously, including the transverse temporal cortex, superior temporal gyrus, temporal pole, and so on, which are related to auditory information processing, phonological encoding, and lexical selection, as well as syntactic and phrase level processing (Walenski et al., 2019). It is interesting to note that some of the subjects told us that the time-reversed speech sounded like a foreign language. This implies that the subjects possibly recruited brain resources to comprehend the audio sequences of the time-reversed speech even though it had no semantic information. For the normal speech, the subjects decoded the meaningful information and carried out high-level cognitive processing, whereas, for the time-reversed speech, brain areas such as the caudal middle frontal gyrus and precuneus cortex were not activated.

**Table 1:** The detected brain network communities under the conditions of natural and time-reversed speech.

**Community**	**Natural speech**	**Time-reversed speech**
1	L_Caudal anterior-cingulate cortex	L_Caudal anterior-cingulate cortex
	R_Caudal anterior-cingulate cortex	R_Caudal anterior-cingulate cortex
	R_Fusiform gyrus	R_Fusiform gyrus
	R_Insula cortex	R_Insula cortex
	L_Pars opercularis	L_Pars opercularis
	L_Pars triangularis	L_Pars triangularis
	L_Posterior-cingulate cortex	L_Posterior-cingulate cortex
	R_Posterior-cingulate cortex	R_Posterior-cingulate cortex
	L_Precentral gyrus	L_Precentral gyrus
	L_Superior temporal gyrus	L_Superior temporal gyrus
	L_Temporal pole	L_Temporal pole
	R_Temporal pole	R_Temporal pole
	L_Transverse temporal cortex	L_Transverse temporal cortex
	R_Transverse temporal cortex	R_Transverse temporal cortex
	L_Caudal middle frontal gyrus	L_Inferior temporal gyrus
	R_Caudal middle frontal gyrus	L_Medial orbital frontal cortex
	L_Cuneus cortex	R_Pars triangularis
	L_Insula cortex	L_Rostral anterior cingulate cortex
	L_Lateral occipital cortex	R_Rostral middle frontal gyrus
	L_Precuneus cortex	
	R_Precuneus cortex	
2	R_Superior temporal sulcus	R_Superior temporal sulcus
	L_Entorhinal cortex	L_Entorhinal cortex
	L_Fusiform gyrus	L_Fusiform gyrus
	R_Inferior parietal cortex	R_Inferior parietal cortex
	L_Middle temporal gyrus	L_Middle temporal gyrus
	R_Middle temporal gyrus	R_Middle temporal gyrus
	R_Parahippocampal gyrus	R_Parahippocampal gyrus
	L_Paracentral lobule	L_Paracentral lobule
	R_Pars opercularis	R_Pars opercularis
	L_Postcentral gyrus	L_Postcentral gyrus
	R_Postcentral gyrus	R_Postcentral gyrus
	R_Precentral gyrus	R_Precentral gyrus
	L_Superior frontal gyrus	L_Superior frontal gyrus
	L_Superior parietal cortex	L_Superior parietal cortex
	R_Superior parietal cortex	R_Superior parietal cortex
	L_Supramarginal gyrus	L_Supramarginal gyrus
	R_Supramarginal gyrus	R_Supramarginal gyrus
	R_Entorhinal cortex	R_Isthmus-cingulate cortex
	R_Inferior temporal gyrus	L_Lateral orbital frontal cortex
	L_Parahippocampal gyrus	R_Lateral orbital frontal cortex
	L_Superior temporal sulcus	R_Paracentral lobule
		L_Pars orbitalis
		R_Rostral anterior cingulate cortex
		L_Rostral middle frontal gyrus
		R_Superior frontal gyrus
		R_Superior temporal gyrus

*The different brain regions in the communities were highlighted*.

**Figure 9:**
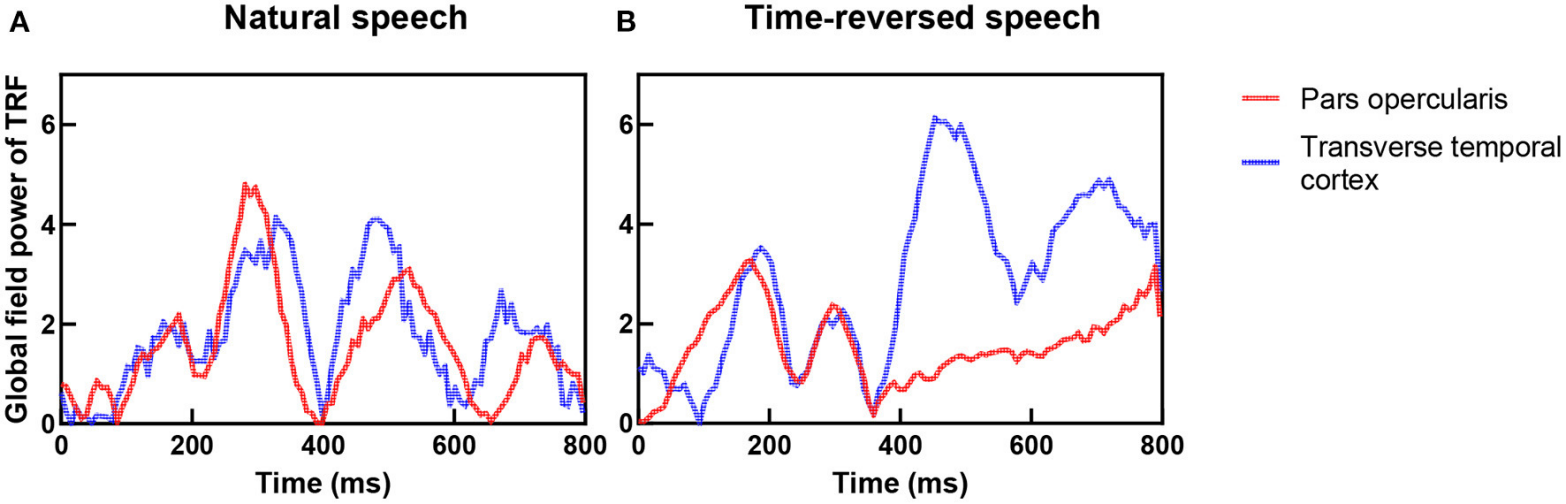
Coupling between frontal area (red color) and auditory cortex (blue color) for natural speech **(A)** and time-reversed speech **(B)**.

In previous research, the coupling of the auditory cortex and frontal areas was reported, and this coupling increases when speech has higher intelligibility (Park et al., 2015). They hypothesized that top-down signals from the frontal brain areas causally modulate the phases of brain oscillations in the auditory cortex. To verify this hypothesis, we checked the TRFs of the pars opercularis in the frontal area and the primary auditory cortex (transverse temporal cortex), and we show them in Figure 9. One can see that the coupling between the frontal area and primary auditory was stronger for the natural speech than the time-reversed speech, where the correlation coefficient was 0.65 for the natural speech but 0.23 for the time-reversed speech. In particular, the coupling rapidly decreased after 400 ms for the time-reversed speech. This may indicate that high-level language brain areas were not recruited in the audio processing for the time-reversed speech because it was not comprehended by the subjects.

**Figure 10:**
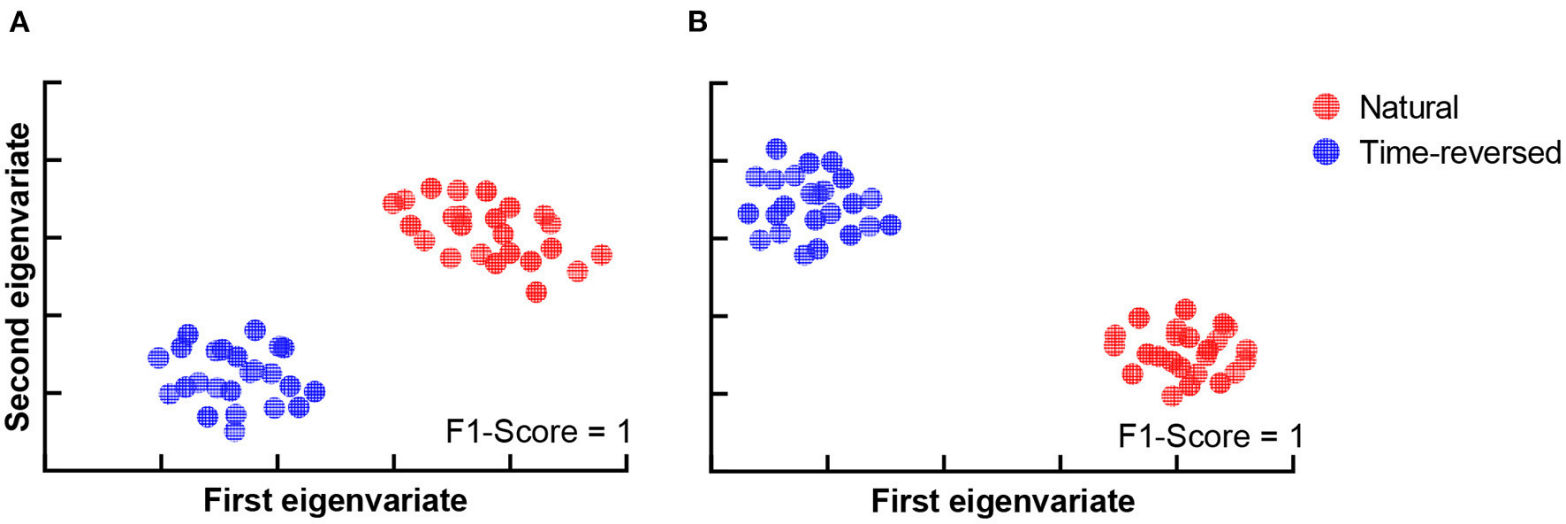
Clustering results for two communities. **(A)** Community 1, **(B)** Community 2.

Furthermore, we attempted to investigate how much the brain networks could be separated on the basis of community detection. The results are shown in Figure 10. The brain networks for the normal and time-reversed speech were clearly separated into two independent clusters for both communities 1 and 2.

## 5. Discussion

### 5.1. Proposed Approach Extends Cognitive Understanding of TRFs to Brain Source Space

TRFs have been used to reflect functional roles for the cognitive processing of speech (Liberto et al., 2015; Broderick et al., 2018), but their estimation is predominantly limited to the electrode/sensor space. The cortical origins of the underlying speech processing are still not clear. Although recent electrocorticography (ECoG) studies provide some exciting results for the cortical origins of speech processing (Anumanchipalli et al., 2019; Zhang et al., 2021), such intracranial electrography is not friendly for healthy people, and it is also hard to investigate the whole-brain distribution of the sources due to its limited spatial range. As the accuracy of EEG source reconstruction has been improved, in this paper, we proposed an approach to extending TRFs from the electrode space to the source space by using a source reconstruction method. Source-based TRFs can reflect the cortical origins of the underlying natural spoken language processes. Then, we used the source-based TRFs to investigate the brain network during speech comprehension on the basis of community detection. The contributions of this paper are as follows. First, we proposed an approach to perform source localization for the single-trial natural speech paradigm. For accurate source localization results, we introduced a functional hyper-alignment method combined with additive averaging over all subjects. From the accuracy of speech envelope prediction, our proposed approach showed good performance. Second, from the clustering results of natural speech and time-reversed speech, the source TRFs based on our method can be used in revealing the cognitive mechanism for natural speech processing. Third, the findings obtained with our approach are highly consistent with previous meta-analysis of natural language processing (see Section More Regions Recruited in Brain Network Communities Under Natural Speech Paradigm). To the best of our knowledge, this is the first study that tries to use community detection to address the EEG-based brain network during natural speech comprehension task. As a result, our approach can be a reasonable way of performing community detection for complex natural speech processing tasks using EEG in the future.

### 5.2. Neural Response to Speech Envelope Reflects High-Level Semantic Processing

In previous research, some contrary results regarding the neural entrainment to speech envelope were reported. For example, the speech envelope is usually considered to be related to low-level acoustic features such as the syllable boundary, while some studies reported that the neural entrainment to speech envelope was stronger when speech was easy to understand (Park et al., 2015; Vanthornhout et al., 2018). They considered a high-level top-down prediction mechanism driving the coupling between neural signal and speech envelope more strongly during intelligible speech perception than unintelligible speech perception. However, some other studies state that there was no difference in the neural entrainment to speech envelope between accessible and inaccessible speech (Howard and Poeppel, 2010; Millman et al., 2015; Zoefel and VanRullen, 2016). Here, we have enough evidence to speculate that the opposite may be caused by unexpected noises in the neural signal. The TRF-based brain network is a kind of supervised network structure. In this study, it is only related to the speech envelope. From the clustering results of Figure 3, the cognitive-level difference between an intelligible speech envelope and unintelligible speech envelope is affected by unexpected noises. However, after the noise reduction by the proposed method, this kind of semantic-driven speech envelope can be separated from non-semantic driven sound. From Figure 4, the TRFs for STS and MTG showed the typical semantic processing components around 400 ms in the natural speech condition, and from the community results, we found that the middle temporal gyri, left inferior frontal gyrus, and auditory cortex are in the same community, which may indicate a top-down prediction mechanism for improving the coupling between neural responses and speech envelopes.

### 5.3. More Regions Recruited in Brain Network Communities Under Natural Speech Paradigm

The natural speech results show less left-lateralization than seen under the traditional paradigm. For over a century, it has been thought that the frontal and temporoparietal regions of the left hemisphere are crucial for speech processes. However, in the natural language paradigm, it has been shown that daily speech comprehension involves bilateral networks, not only left-lateralization in traditional studies (Jung-Beeman, 2005; Huth et al., 2016; Tang et al., 2017; Hamilton et al., 2018). The natural language paradigm reveals more widespread responses to the speech comprehension process, not only to language-specific areas such as Wernicke's and Broca's (Lerner et al., 2011; Simony et al., 2016).

**Figure 11:**
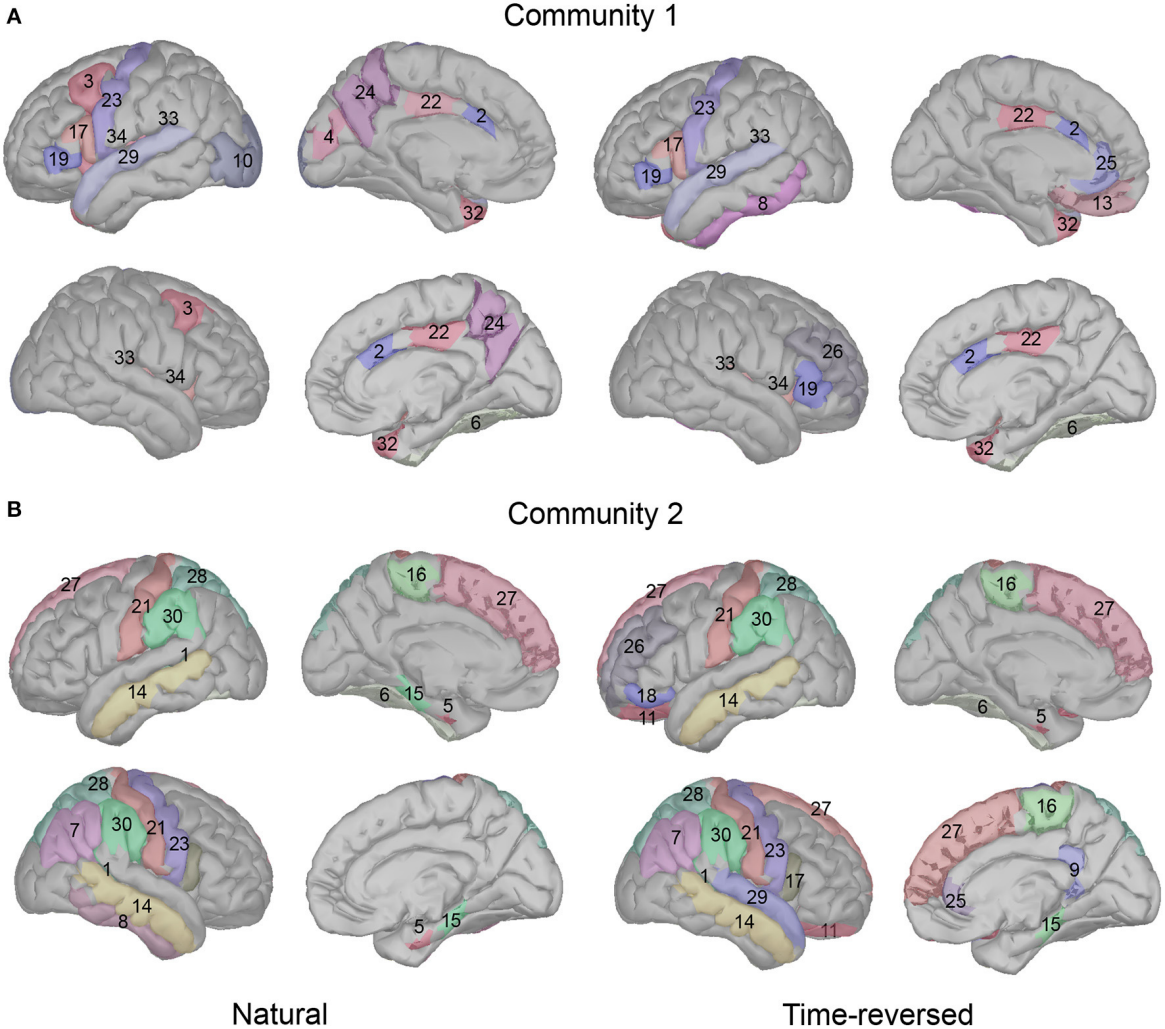
Cortical regions in Desikan-Killiany cortical atlas for community 1 **(A)** and community 2 **(B)**.

On the basis of our results, we see a high consistency with previous speech processing research. We illustrate our community results for natural speech in Figure 11, which references the DKA surface. One can see that the brain areas that are activated in community 1 include the primary auditory cortex (transverse temporal), inferior frontal gyrus (IFG, which includes pars opercularis and pars triangularis), and insula cortex, which are related to phrase structure building and lexical selection, the middle frontal gyrus and precentral gyrus, which are related to phonological decoding, the temporal pole, which is related to semantic processing, the superior temporal gyrus, which is related to phonological encoding, word recognition, and syntactic processing, and the cingulate cortex, which is related to sentence comprehension (see meta-analysis in Walenski et al., 2019). In addition to these areas, the occipital cortex is also activated. Although the task in our experiment is speech perception, some studies have reported the importance of natural speech perception in the visual cortex (Micheli et al., 2020; Brandman et al., 2021).

In addition, long-time story comprehension also involves various cognitive processes such as memory, attention, and information integration. In our case, the precuneus, posterior-cingulate cortex (PCC), prefrontal cortex, and temporal pole are the main brain areas of the default mode network (DMN), which accumulates and integrates information over hundreds of seconds with our intrinsic information of memories during story perception (Simony et al., 2016; Yeshurun et al., 2021). And the activation in the fusiform gyrus, insula cortex, superior temporal gyrus, temporal pole, and lateral occipital cortex may be related to auditory attention process, because the similar regions are reported in a previous auditory attention task (Alho et al., 2015).

In community 2, the brain areas that were activated included the middle temporal gyrus and fusiform cortex, which are related to lexical-semantic processing, the superior frontal gyrus (supplementary motor area), which is related to effortful comprehension and phonological decoding, and the supramarginal gyrus and parahippocampal gyrus, which are related to sentence-level processing. The parietal cortex is considered to be involved in both auditory and visual sentence comprehension. Additionally, the banks of the superior temporal sulcus and postcentral gyrus are important in complex sentence-level processing (Vigneau et al., 2006; Matchin and Hickok, 2016). Some research has reported that the paracentral lobule is activated more in 3-year-old than toddlers in the speech perception task, and it is pointed out that this area is important for language acquisition (Redcay et al., 2008). In addition, the entorhinal cortex is considered to be a high-level brain area for speech perception, and it has a direct connection with the auditory cortex; however, deafness may alter the brain's connectivity between the auditory cortex and entorhinal cortex (Kral et al., 2016).

We compared the difference in the community results between the natural and time-reversed speech. The activation for natural speech in community 1 was mainly the brain areas for the auditory attention (Salmi et al., 2009; Alho et al., 2015). However, in the time-reversed case, subjects hardly paid attention to the unintelligible speech the whole time without any positive top-down feedback. Therefore, the attention related regions were not activated in the time-reversed condition. Because of top-down processing, subjects were easily able to predict the following words or contexts for the natural speech, they used fewer brain resources for processing the upcoming speech stream, and only few auditory brain areas were activated for the natural speech condition in community 2. On the contrary, subjects used more brain resources for processing the time-reversed speech, especially in the frontal brain areas in the left hemisphere and even more brain areas in the right hemisphere.

## 6. Conclusion

In this paper, we proposed a functional hyper-alignment method with the additive average method to reduce the noises caused by individual physiological activities and/or electrode settings for subjects. Instead of using raw EEG signals, we reconstructed brain sources for estimating the temporal response functions in order to be able to study the brain networks underlying natural speech comprehension. The preliminary brain network was the pairwise connection of the brain areas, where links were defined by the correlation coefficient of the TRFs between paired areas. A multi-scale community detection was applied to the preliminary brain networks obtained from a natural speech comprehension task and time-reversed speech processing task to explore functional brain network communities. The results showed two clearly distinguishable functional network communities for semantically driven speech processing and for non-semantically driven (time-reversed speech) audio processing. The functional brain network can be explained on the basis of the achievements of past research. It was also verified that the multi-scale community detection method is suitable for source reconstruction-based brain network studies.

## Data Availability Statement

The datasets generated for this study are available on request to the corresponding author.

## Ethics Statement

The studies involving human participants were reviewed and approved by Research Ethics Committee of Tianjin University. The patients/participants provided their written informed consent to participate in this study.

## Author Contributions

DZ performed the experiments, analyzed the data, and prepared the manuscript. GZ inspired the research idea and revised the manuscript. JD interpreted the results of the experiments and revised the manuscript. MU provided the code for extracting the speech feature based on cochlear filterbank and revised the manuscript. XL provided the idea and programmed the code for community detection. All authors approved the final version of the manuscript for submission.

## Funding

This research was supported by the National Natural Science Foundation of China (No.61876126), a Grant-in-Aid for Scientific Fund for the Promotion of Joint International Research [Fostering Joint International Research (B); 20KK0233], and in part by JSPS KAKENHI Grant (20K11883).

## Conflict of Interest

The authors declare that the research was conducted in the absence of any commercial or financial relationships that could be construed as a potential conflict of interest.

## Publisher's Note

All claims expressed in this article are solely those of the authors and do not necessarily represent those of their affiliated organizations, or those of the publisher, the editors and the reviewers. Any product that may be evaluated in this article, or claim that may be made by its manufacturer, is not guaranteed or endorsed by the publisher.
